# When to call it off: defining transplant candidacy limits in liver donor liver transplantation for hepatocellular carcinoma

**DOI:** 10.1186/s12885-020-07238-w

**Published:** 2020-08-12

**Authors:** Abu Bakar Hafeez Bhatti, Ammal Imran Qureshi, Rizmi Tahir, Faisal Saud Dar, Nusrat Yar Khan, Haseeb Haider Zia, Shahzad Riyaz, Atif Rana

**Affiliations:** 1 Division of Hepato-Pancreatico-Biliary Surgery and Liver Transplantation, Shifa International Hospital, Islamabad, Pakistan; 2 Division of Transplant Hepatology, Shifa International Hospital, Islamabad, Pakistan; 3 Division of Radiology, Shifa International Hospital, Islamabad, Pakistan

**Keywords:** Microvascular invasion, AFP, Recurrence, UCSF criteria, Liver transplantation

## Abstract

**Background:**

Living donor liver transplantation (LDLT) is an acceptable treatment option for hepatocellular carcinoma (HCC). Traditional transplant criteria aim at best utilization of donor organs with low risk of post transplant recurrence. In LDLT, long term recurrence free survival (RFS) of 50% is considered acceptable. The objective of the current study was to determine preoperative factors associated with high recurrence rates in LDLT.

**Methods:**

Between April 2012 and December 2019, 898 LDLTs were performed at our center. Out of these, 242 were confirmed to have HCC on explant histopathology. We looked at preoperative factors associated with ≤ 50%RFS at 4 years. For survival analysis, Kaplan Meier curves were used and Cox regression analysis was used to identify independent predictors of recurrence.

**Results:**

Median AFP was 14.4(0.7–11,326.7) ng/ml. Median tumor size was 2.8(range = 0.1–11) cm and tumor number was 2(range = 1–15). On multivariate analysis, AFP > 600 ng/ml [HR:6, CI: 1.9–18.4, *P* = 0.002] and microvascular invasion (MVI) [HR:5.8, CI: 2.5–13.4, *P* <  0.001] were independent predictors of 4 year RFS ≤ 50%. When AFP was > 600 ng/ml, MVI was seen in 88.9% tumors with poor grade and 75% of tumors outside University of California San Francisco criteria. Estimated 4 year RFS was 78% for the entire cohort. When AFP was < 600 ng/ml, 4 year RFS for well-moderate and poor grade tumors was 88 and 73%. With AFP > 600 ng/ml, RFS was 53% and 0 with well-moderate and poor grade tumors respectively (*P* <  0.001).

**Conclusion:**

Patients with AFP < 600 ng/ml have acceptable outcomes after LDLT. In patients with AFP > 600 ng/ml, a preoperative biopsy to rule out poor differentiation should be considered for patient selection.

## Background

Liver transplantation is an established treatment modality in patients with hepatocellular carcinoma (HCC) and cirrhosis [1, 2]. Milan criteria and University of California San Francisco (UCSF) criteria are the most widely accepted transplant criteria for patient selection [3, 4]. These criteria have been primarily derived in the deceased donor liver transplant (DDLT) setting and aim at best utilization of donor organs. In living donor liver transplantation (LDLT), issues such as prolonged waiting times and competition for donor organs are not encountered. Thus, Milan and UCSF criteria appear restrictive. As a result, most LDLT centers in Asia perform LDLT for HCC outside these criteria [5].

The minimum acceptable recurrence free survival(RFS) in the setting of LDLT remains debatable. Considering the operative risk to a living donor in LDLT, long term RFS of 50% is considered the benchmark [6, 7]. Other than tumor size and number; certain factors like AFP level, microvascular invasion (MVI) and poor grade have a significant impact on post transplant recurrence [8, 9]. With a keen desire to increase patient pool eligible for transplantation, there is a need to identify patients who should be denied LDLT due to unacceptable recurrence risk.

We are a high volume LDLT center, and perform a significant number of transplants for HCC; some of whom are outside traditional transplant criteria. This provides a unique opportunity to assess outcomes in advanced tumors managed with LDLT.

In the setting of LDLT, long term RFS < 50% should be considered unacceptable and transplant in such patients should be considered futile. The objective of the current study was to identify preoperative factors, in the presence of which, LDLT can be potentially declined.

## Methods

Between April 2012 and December 2019, a total of 898 living donor liver transplants were performed at our center. For this study we included adult patients with a diagnosis of HCC on explant histopathology who underwent LDLT between April 2012 and June 2019 (*n* = 242).

Details of diagnostic workup and patient selection for transplantation have been reported elsewhere [10, 11]. The diagnosis of HCC was made on a liver dynamic CT scan. A dynamic MRI scan of the liver was performed if CT scan findings were not conclusive. Biopsy was reserved for cases where diagnosis could not be established on dynamic imaging. All patients were discussed in multi disciplinary team meeting. Extra-hepatic metastasis and main portal vein tumor thrombosis were considered absolute contraindications for transplantation. Preoperative treatments including trans-arterial chemoembolization (TACE), radio-frequency ablation (RFA) and percutaneous ethanol injection (PEI) were discussed with all patients. With accumulating experience, patients with tumor size > 10 cm, segmental or lobar portal vein tumor thrombus, or AFP > 1000 ng/ml were considered for down-staging, if feasible. In case there was an anticipated delay, these treatments were used as a bridge for transplantation.

We looked at patient demographics, AFP levels, etiology of liver failure, Model for end stage liver disease(MELD), and Child Turcot Pugh (CTP) scores, tumor variables, and preoperative treatment. Prognostic variables including Milan and UCSF criteria for transplantation, tumor grade, microvascular invasion, AFP, MELD and CTP score, graft to recipient weight ratio (GRWR), and preoperative treatment were assessed to determine impact on RFS. We used different AFP cutoff values for prognostication based on previous reports [11–13]. We classified 4 year RFS > 50% as acceptable while ≤50% as unacceptable RFS based on previous recommendations [6, 7].

Primary objective of the study was to identify preoperative factors associated with unacceptable recurrence rates in the setting of LDLT. The RFS was calculated by subtracting date of recurrence from date of transplantation. Univariate analysis was performed using Kaplan Meier survival curves and Log rank test was used to determine significance. Variables that were associated with RFS < 50% at 4 years were included in the multivariate Cox proportional hazard model. To determine AFP cutoff for recurrence, we used receiver operator curves (ROC). To increase transplant eligible patients, we aimed for high specificity on ROC curves as previously shown [14]. Prognostic groups were developed based on preoperative prognostic factors associated with < 50% RFS at 4 years. Chi square and Fischer test were used for categorical variables. A *P* value < 0.05 was considered statistically significant. All analysis was performed on Statistical package for the social sciences (SPSS version 20). The study was approved by the hospital ethics committee.

## Results

### Patient characteristics

Median age was 53(30–74) years. Median BMI was 25.2(15.4–40) kg/m^2^. Median AFP was 14.4(0.7–11,326.7) ng/ml. Median tumor size on explant was 2.8(range = 0.1–11) cm. Mean number of tumor nodules was 2(range = 1–15). Prior HCV and HBV exposure was present in 193 (79.7%) and 49(20.2%) patients respectively as shown in Table 1. Pre transplant TACE and/or RFA was performed in 56(23.1%) patients.

**Table 1:** Patient characteristics

	Number (***N*** = 242)	Percent (%)
**Gender**		
Males	203	83.9
**Age group**		
≤ 40	14	5.8
40–50	80	33
50–60	113	46.7
> 60	35	14.5
**Etiology**		
HCV	183	75.6
HBV	34	14
HCV + HBV	10	4.1
HBV + HDV	5	2
Cryptogenic	7	2.9
Others	3	1.2
**Graft to recipient weight ratio**		
< 0.8	40	16.5
> 0.8	202	83.5
**MELD score**		
≤ 10	27	11.2
11–20	129	53.3
21–30	77	31.8
> 31	9	3.7
**CTP class**		
A	50	20.7
B	100	41.3
C	92	38
**Extent**		
Within Milan	134	55.4
Within UCSF criteria	16	6.6
Outside UCSF criteria	92	38
**Tumor nodules**		
1	118	48.7
2	56	23.1
3	18	7.4
Multiple	50	20.7
**AFP (ng/ml) (*N*** **= 232)**		
Normal	74	31.9
≤ 600	136	58.6
> 600	22	9.5
**Preoperative treatment**		
TACE	54	22.3
RFA	11	4.5
**Grade**		
Well/moderate	163	67.3
Poor	71	29.4
Complete response	8	3.3
**Micro vascular invasion**		
Present	85	35.1

### Prognostic factors associated with inferior 4 year RFS

**Fig. 1:**
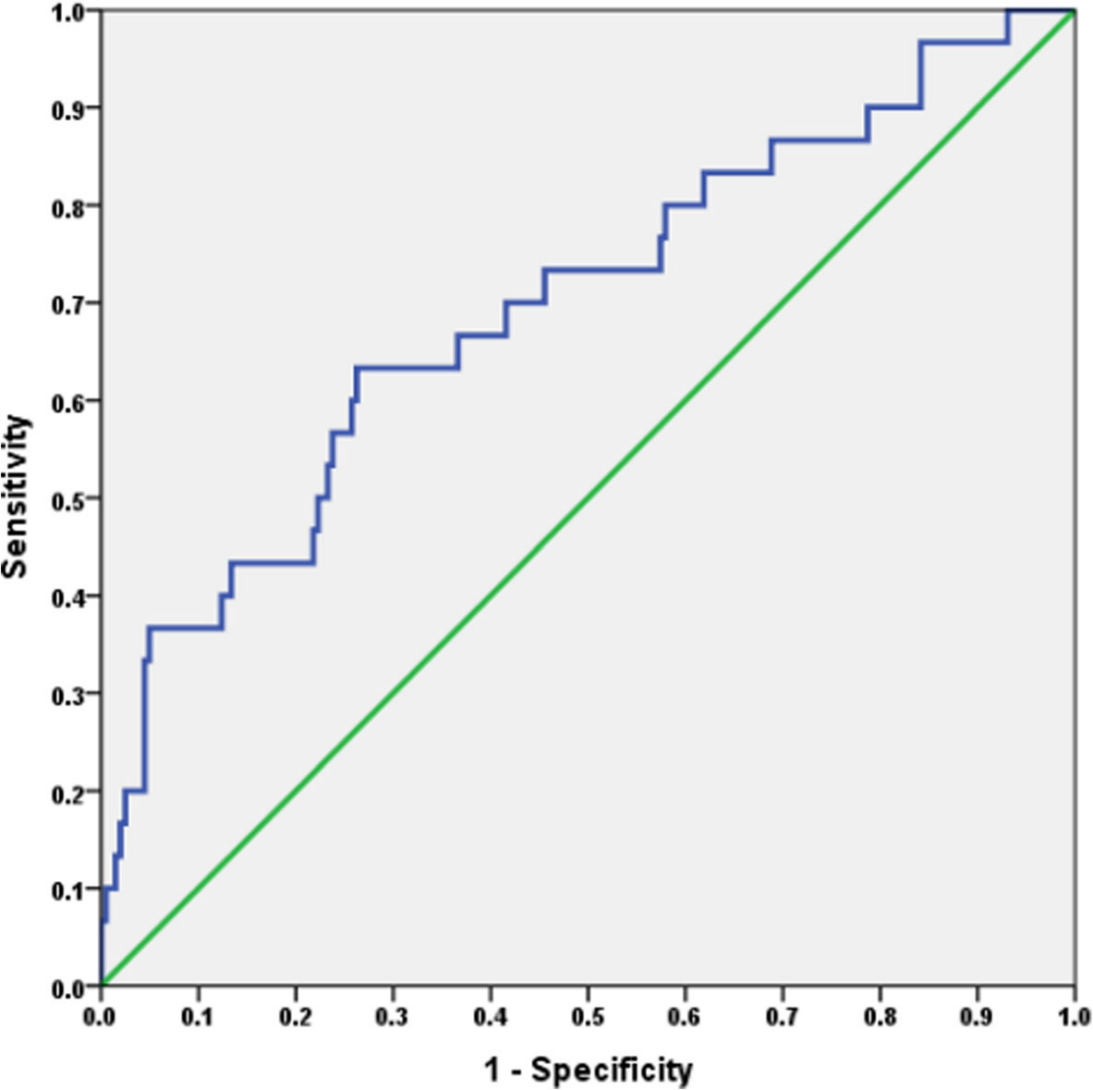
ROC curve for AFP and HCC recurrence

Estimated 4 year RFS was 78% for the entire study cohort. Table 2 demonstrates 4 year RFS for various prognostic factors. An AFP cutoff of 40 ng/ml had sensitivity of 64% and specificity of 74% for recurrence. While AFP cutoff of 600 ng/ml had a sensitivity of 35% and specificity of 97% for recurrence (AUC = 0.69, *P* < 0.0001)(Fig. 1). AFP level and MVI were associated with < 50% RFS. The 4 year RFS was 30% versus 83% (*P* < 0.001), 45% versus 79% (*P* < 0.001) and 41% versus 78% (*P* < 0.001) for AFP cutoffs of 600 ng/ml, 1000 ng/ml and 2000 ng/ml. In patients with MVI, 4 year RFS was 49% versus 90%(*P* < 0.001).

**Table 2:** Prognostic factors for estimated 4 year recurrence free survival

Prognostic factors		Recurrence free survival (%)	***P*** value
**Milan criteria**	In	89	< 0.0001
	out	53	
**UCSF criteria**	In	85	0.02
	out	54	
**Number of tumors**	≤3	82	0.2
	> 3	46	
**Size of largest tumor (cm)**	≤ 5	82	0.004
	> 5	62	
**AFP (ng/ml)**	< 600	83	< 0.0001
	> 600	30	
	< 1000	79	< 0.0001
	> 1000	45	
	< 2000	78	< 0.0001
	> 2000	41	
**Grade**	Well/moderate	85	0.008
	poor	61	
**Microvascular invasion**	Absent	90	< 0.0001
	present	49	
**Pretransplant treatment**	Not received	86	0.01
	received	54	
**Graft to recipient weight ratio**	> 0.8	80	0.1
	< 0.8	74	
**CTP class**	A	82	0.3
	B	73	
	C	84	
**MELD score**	< 20	80	0.6
	> 20	77

**Fig. 2:**
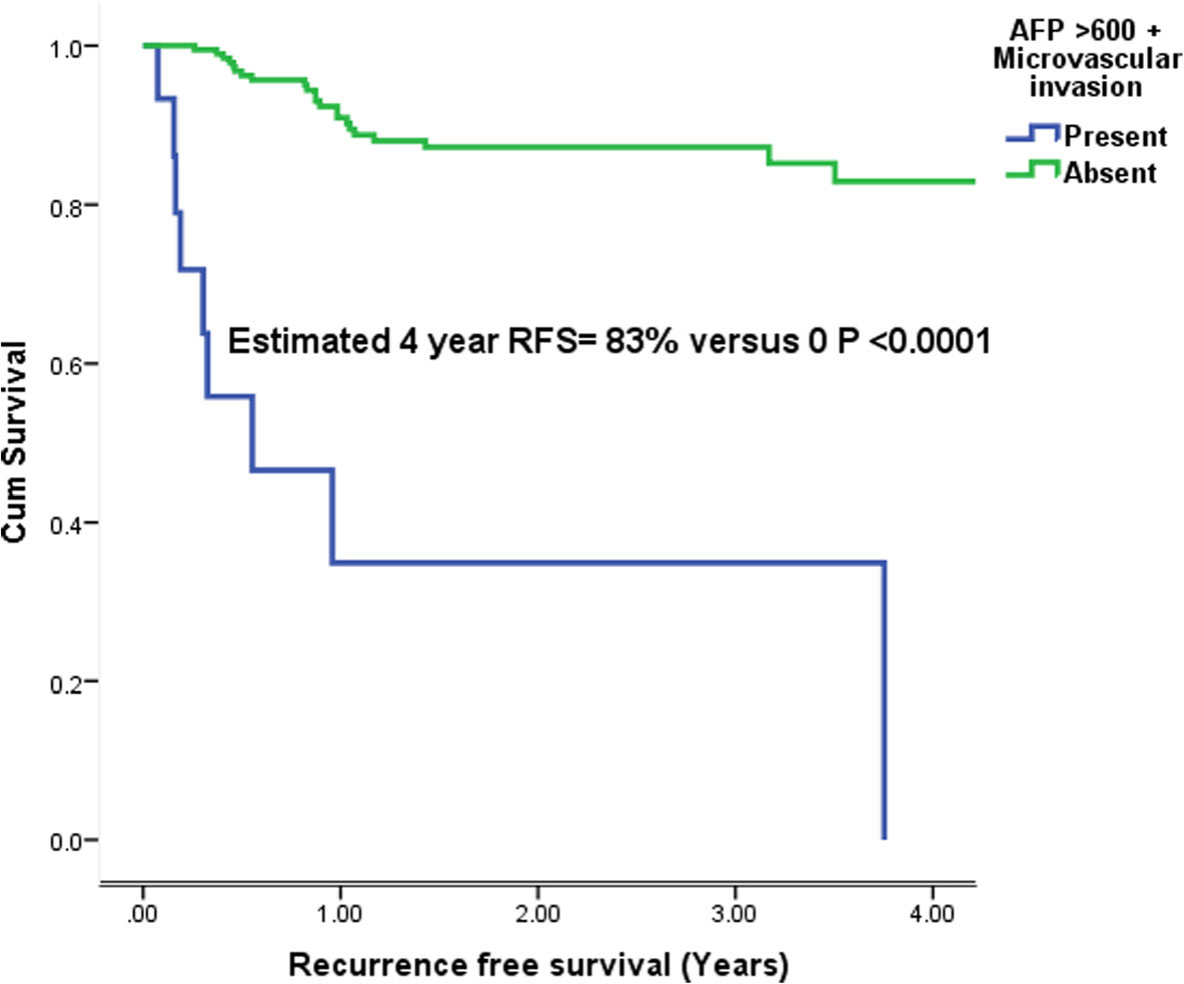
Estimated 4 year recurrence free survival in patients with combined AFP > 600 ng/ml and microvascular invasion

A multivariate analysis including prognostic factors associated with 4 year RFS < 50% was performed as shown in Table 3. An AFP > 600 ng/ml and MVI were independent predictors of RFS. Risk of recurrence was significantly increased with AFP > 600 ng/ml [HR:6, CI: 1.9–18.4, *P* = 0.002] and MVI [HR:5.8, CI: 2.5–13.4, *P* < 0.001].The 4 year RFS in patients with combined AFP > 600 ng/ml and MVI was 0 versus 83% (*P* < 0.001) as shown in Fig. 2.

**Table 3:** Multivariate analysis of risk factors associated with 4 year recurrence free survival < 50% on univariate analysis

	Multivariate analysis		
	Hazard ratio	Confidence interval	***P*** value
**AFP (ng/ml)**			
< 600	1	1.9–18.4	0.002
> 600	6		
**AFP (ng/ml)**			
< 1000	1	0.4–13	0.32
> 1000	2.3		
**AFP (ng/ml)**			
< 2000	1	0.59–16.4	0.19
> 2000	3.1		
**Microvascular invasion**			
absent	1	2.5–13.4	< 0.0001
present	5.8

### Preoperative factors associated with MVI

Since MVI cannot be accurately determined preoperatively, we looked for factors associated with high risk (> 50%) of MVI. When combined with AFP > 600 ng/ml, tumors outside UCSF criteria and poor grade were significant factors associated with high risk of MVI as shown in Table 4.

**Table 4:** Rates of microvascular invasion with various prognostic variables

	Microvascular invasion present		Microvascular invasion absent		
	Number	Percent	Number	Percent	*P* value
AFP > 600 ng/ml	15	68.1	7	31.9	0.001
Poor grade	38	54.2	32	45.8	< 0.0001
Tumor size > 5 cm	28	63.6	16	36.4	< 0.0001
Tumor size > 6.5 cm	15	75	5	25	< 0.0001
Tumor number > 3	28	56	22	44	0.001
Tumors outside Milan criteria	54	52.9	48	47.1	< 0.0001
Tumors outside UCSF criteria	48	55.8	38	54.2	< 0.0001
**MVI with each variables when AFP > 600 ng/ml**					
Poor grade	8	88.9	1	11.1	< 0.0001
Tumor size > 6.5 cm	3	75	1	25	0.1
Tumor number > 3	5	71.4	2	28.6	0.05
UCSF out tumors	9	75	3	25	< 0.0001

Since AFP and MVI were the only independent predictors of < 50% RFS on multivariate analysis, we developed prognostic groups associated with high risk of MVI based on AFP > 600 ng/ml. The highest risk of MVI was seen in patients with AFP > 600 ng/ml and poor grade (88.9%) and AFP > 600 ng/ml/UCSF out tumors (75%) as shown in Table 5.

**Table 5:** Risk of Microvascular invasion based on AFP, UCSF criteria and poor grade prognostic groups

	Microvascular invasion present		Microvascular invasion absent		
	Number	Percent	Number	Percent	*P* value
*Group 1*					
AFP > 600 + UCSF out	9	75	3	25	< 0.001
AFP > 600 + UCSF in	6	60	4	40	
AFP < 600 + UCSF out	39	53.4	34	46.6	
AFP < 600 + UCSF in	31	22.1	109	77.9	
*Group 2*					
AFP > 600 + poor diff	8	88.9	1	11.1	< 0.001
AFP > 600 - poor diff	7	53.9	6	46.1	
AFP < 600 + poor diff	30	50.8	29	49.2	
AFP < 600 - poor diff	40	26.5	111	73.5	

### Patient selection for LDLT

**Fig. 3:**
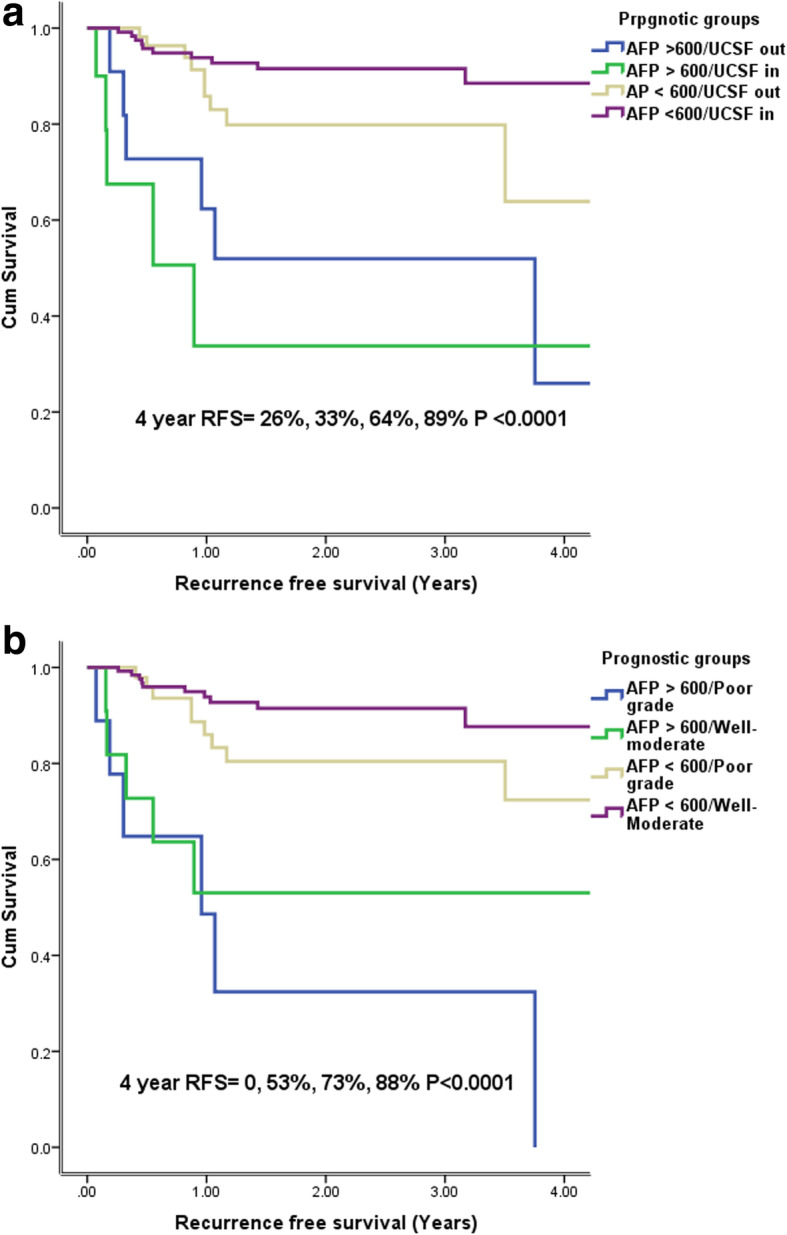
**a** Estimated 4 year recurrence free survival based on AFP and UCSF criteria **b** based on AFP and tumor grade

Table 6 demonstrates actual recurrence rates in various prognostic groups. All patients with AFP < 600 ng/ml within and outside UCSF criteria, irrespective of tumor grade, had an acceptable 4 year RFS (> 50%) as shown in Fig. 3. The estimated 4 year RFS in patients with AFP > 600 ng/ml irrespective of whether tumors were within or outside UCSF criteria was < 50% (Fig. 3a). In patients with AFP > 600 ng/ml, 4 year RFS was 53% for well-moderately differentiated tumors while it was 0 and not reached with poorly differentiated tumors (Fig. 3b). When patients with AFP > 600 ng/ml and poor differentiation were excluded, the 4 year RFS of our patient cohort was 82%.

**Table 6:** Recurrence in prognostic groups based on AFP, UCSF criteria and tumor grade

	Recurrence		No Recurrence			
	Number	Percent	Number	Percent	Total	***P*** value
*Group 1*						
AFP > 600 + UCSF out	6	50	6	50	12	< 0.001
AFP > 600 + UCSF in	5	50	5	50	10	
AFP < 600 + UCSF out	11	13.5	70	86.5	81	
AFP < 600 + UCSF in	8	6.2	121	93.8	129	
*Group 2*						
AFP > 600 + poor diff	6	66.7	3	33.4	9	< 0.001
AFP > 600 - poor diff	5	38.4	8	61.6	13	
AFP < 600 + poor diff	9	15.2	50	84.8	59	
AFP < 600 - poor diff	10	6.6	141	93.4	151

## Discussion

The current study reports outcomes of a significantly large cohort of patients who underwent LDLT for HCC [9, 14]. A small percentage of patients (< 10%)had AFP > 600 ng/ml. This group was further assessed for poor differentiation. Overall, < 5% patients had AFP > 600 ng/ml with poor differentiation. We believe this is the group of patients with very high risk of recurrence and needs to be identified on preoperative biopsy.

We identified AFP and MVI as important prognostic variables for recurrence post transplantation. The prognostic impact of AFP and MVI is well known [8, 14–19]. The challenge remains in preoperatively identifying patients likely to have MVI. Various imaging modalities including computed tomography (CT), magnetic resonance imaging(MRI), positron emission tomography (PET) scan and tumor marker cutoffs have been assessed but remain inconsistent in detection of MVI [18–21].Preoperative biopsy is also not accurate in detecting MVI but carries sinister risk of tumor seeding [18]. This limits the widespread application of preoperative biopsy in transplant candidates and a cautious approach is warranted. Based on results of the current study, only 22/242(9%) patients would mandate a preoperative biopsy. Poor grade in this group would be a surrogate marker of MVI as 88.9% patients had MVI when AFP > 600 ng/ml and poor grade were present together.

Role of preoperative biopsy in the diagnosis of HCC, in particular before transplantation remains less clear. The obvious advantage includes pertinent diagnostic and prognostic information [22]. It has been shown that the diagnostic sensitivity and specificity of needle biopsy is 94 and 100% respectively [23]. The drawbacks include an invasive procedure, with risks of bleeding and tumor seeding, and inaccurate information. In recent times, with technical improvements, the risk of tumor seeding (0.001%) and major complications appears (0.004%) to have tremendously reduced and diagnostic errors are rare [24]. Moreover, better knowledge of molecular and immuno histochemical properties of HCC has led to renewed interest in the role of biopsy in patients with HCC [22, 25]. Poor grade has been used by the Toronto and Hangzhou group to select patients unlikely to benefit from transplantation [8, 17]. With Toronto criteria, 108/242(44.6%) patients in the current study would require preoperative biopsy. With AFP > 600 ng/ml as the only indication, irrespective of tumor size and number, we have potentially limited preoperative biopsy to very few patients, increased the number of patient pool eligible for transplantation, and identified a subgroup which despite AFP > 600 ng/ml can be transplanted with acceptable risk of recurrence. In this group, LDLT should only be offered if well to moderate grade is confirmed preoperatively. A strict tumor size or number cutoff to select patients for preoperative biopsy can miss out on patients with high AFP and poor grade leading to unacceptable recurrence risk. We suggest that if poor grade is present in patients with AFP > 600 ng/ml, LDLT should be potentially declined.

In recent years, increasingly complex criteria incorporating tumor size and number, yet relying heavily on biological factors have been proposed [26–29]. These are primarily based on DDLT experience and attempt to increase eligible transplant pool without compromising outcomes when compared with Milan criteria. However, it has been suggested that post transplant outcomes in patients who fulfill these criteria are not comparable to Milan criteria [28]. In the current study, we have attempted to identify eligible candidates with acceptable (> 50%) RFS. The purpose is not achieve outcomes comparable to Milan criteria but to identify all patients who are eligible transplant candidates. This is more relevant to LDLT where there is no obvious benefit of comparing outcomes with non-HCC patients on the waiting list for liver transplantation. Moreover, we have done so using simple well established pretransplant variables that are easily available and applicable.

Worldwide, transplant criteria for HCC are becoming more inclusive, dynamic and biology driven [26, 30–34]. To improve identification of high risk HCC patients for LDLT, some centers have used des-gamma-carboxy prothrombin (DCP) and PET scan. Imaging modalities such as PET scans and tumors markers other than AFP still need validation in terms of their clinical applicability. It is important that patients with HCC in whom LDLT is essentially futile are identified using simpler models with easily applicable tools that have been previously validated to impact prognosis.

## Conclusion

The current study uses well established preoperative variables in a large cohort of HCC patients who underwent LDLT, to identify patients at high risk of post transplant recurrence. Judicious use of preoperative biopsy in patients with AFP > 600 ng/ml can identify patients not suitable for transplantation. We believe, it is more relevant to LDLT setting, where liberal cutoffs are used on tumor dimensions, waiting time is short and competition for donor organs is absent. These results need to be validated in similar settings with longer follow up to determine applicability of current findings.

## Data Availability

The datasets used during the current study are available from the corresponding author on reasonable request.

## Abbreviations

AFP: Alpha fetoprotein
DDLT: Deceased donor liver transplantation
HCC: Hepatocellular carcinoma
LDLT: Living donor liver transplantation
MELD: Model for end stage liver disease
MVI: Microvascular invasion
NLR: Neutrophil to lymphocyte ratio
RFA: Radio frequency ablation
MWA: Microwave ablation
RFS: Recurrence free survival
TACE: Trans arterial chemo embolization
UCSF: University of California San Francisco
